# Vitamin D deficiency and cardiovascular autonomic neuropathy in patients with type 1 diabetes mellitus

**DOI:** 10.1186/1758-5996-7-S1-A44

**Published:** 2015-11-11

**Authors:** Denisson Dias da Silva, Liter William Pinheiro Nunez, Danielle Dias da Silva Pinheiro, Luciana Lobato de Oliveira, Suzanny Silva Ladeira, Natércia Neves Marques de Queiroz, Ana Carolina dos Santos Pinto, Luciana Marques da Costa, Ana Carolina Contente Braga de Souza, Franciane Trindade Cunha de Melo, Karem Miléo Felício, Camila Cavalcante Koury, João Felício Abrahão Neto, Carolina Tavares Carvalho, Thaís Pontes Arbage, Hana Andrade de Rider Brito, Antônio Bentes Figueiredo, Fabrício de Souza Resende, João Soares Felício

**Affiliations:** 1 Hospital Universitário João de Barros Barreto, Belém, Brazil

## Background

Low levels of vitamin D have been suggested to have a negative effect on the pancreatic β cell function, being associated with insulin resistance and an increase in inflammatory markers in diabetic patients. However, the relationship between vitamin D levels and diabetes microvascular complications has not been well established yet.

## Objective

This study aimed to evaluate the association between the levels of 25-hydroxyvitamin D [25(OH)D] and the presence of cardiovascular autonomic neuropathy (CAN) in patients with type 1 Diabetes Mellitus (T1D).

## Materials and methods

We performed a cross-sectional study including 50 patients with T1D, submitted to the dosage of 25(OH)D levels by chemiluminescence and to computerized autonomic tests for the evaluation of CAN. The severity of CAN was graduated in absent, incipient or established.

## Results

Our results showed a correlation of 25(OH)D levels with the presence and severity of CAN in patients with T1D. Patients with established CAN presented lower levels of 25(OH)D in comparison to those without CAN (21.5±7.9 vs 31.5±11.3 ngmL, respectively; p<0.05). Evaluating the severity of CAN, we observed lower levels of 25(OH)D in patients with established CAN compared to those with incipient CAN or absence of CAN (18.6±6.4 vs 31.5±11.3 ngmL, respectively; p<0.05). Additionally, the levels of 25(OH)D were correlated with CAN through 6 of the 7 parameters used in the diagnosis: FMB (r=0.28, p<0.05), FB (r=0.33, p<0.01), FA (r=0.35, p<0.01), Valsalva maneuver (r=0.36, p.0.01), 30: 15 coefficient (r=0.38, p<0.01) and respiratory coefficient (r=0.31, p<0.05).

## Conclusion

This is the first study suggesting the association between 25(OH)D levels with the prevalence and severity of CAN in T1D patients. Further studies are necessary to establish whether vitamin D supplementation could influence the progression of CAN in these patients.

